# Imaging assessment of calcinosis in juvenile dermatomyositis: a narrative review

**DOI:** 10.1186/s12969-025-01098-z

**Published:** 2025-04-25

**Authors:** Belina Y. Yi, Jessica Perfetto, Evin Rothschild, Kelly Rouster-Stevens, Amanda Robinson, Kathryn Cook, Delaney D. Ding, Andrea Eagle Child, Ovgu Kul Cinar, Barbara Limbach, Charalampia Papadopoulou, Lesley Ann Saketkoo, Adam Schiffenbauer, Heinrike Schmeling, Antonia Valenzuela, Susan Shenoi, Dawn M. Wahezi, for the CARRA JDM Calcinosis workgroup

**Affiliations:** 1 Johns Hopkins University School of Medicine, Baltimore, MD USA; 2 Hassenfeld Children’s Hospital at NYU Langone, New York, NY USA; 3 Emory University, Children’s Healthcare of Atlanta, Atlanta, GA USA; 4 University of Utah, Salt Lake City, UT USA; 5 Akron Children’s Hospital, Akron, OH USA; 6 University of Florida College of Medicine, Gainesville, FL USA; 7 Childhood Arthritis and Rheumatology Research Alliance (CARRA), Calgary, AB Canada; 8 Great Ormond Street Hospital for Children NHS Foundation Trust, London, UK; 9 Cure JM, Childhood Arthritis and Rheumatology Research Alliance (CARRA), Wolcottville, IN USA; 10 New Orleans Scleroderma and Sarcoidosis Patient Care and Research Center, Louisiana State University and Tulane University Medical Schools, New Orleans, LA USA; 11 National Institute of Environmental Health Sciences (NIEHS), National Institutes of Health (NIH), Bethesda, MD USA; 12 Section of Rheumatology, Department of Pediatrics, Cumming School of Medicine, University of Calgary, Calgary, AB Canada; 13 Department of Clinical Immunology and Rheumatology, Pontificia Universidad Católica de Chile, Santiago, Chile; 14 Seattle Children’s Hospital and Research Center, University of Washington, Seattle, USA; 15 Children’s Hospital at Montefiore, Bronx, NY USA

**Keywords:** Calcinosis, Juvenile dermatomyositis, Idiopathic inflammatory myopathy, x-ray, Computed tomography, Magnetic resonance imaging, Ultrasound

## Abstract

Calcinosis is a severe manifestation of juvenile and adult idiopathic inflammatory myopathies, which can lead to pain, limited range of motion, disfigurement, and infection. It is more common in juvenile idiopathic inflammatory myopathies, especially in juvenile dermatomyositis (JDM). Calcinosis can be visible on cutaneous surfaces, although can also present in muscles and internal organs, making a thorough assessment difficult without imaging modalities. In this narrative review, we discuss different imaging modalities used in evaluating JDM-associated calcinosis including X-ray, computed tomography (CT), magnetic resonance imaging (MRI), and ultrasound (US).

## Background

Calcinosis is formation of calcium deposits in the skin, subcutaneous tissue, fascia, and/or muscles, and is a complication of various autoimmune connective tissue diseases such as idiopathic inflammatory myopathies (IIM) and scleroderma. IIM are multi-organ systemic diseases characterized by chronic inflammation of striated muscle and/or skin, and can involve internal organs such as lungs and gastrointestinal systems. Calcinosis is a common manifestation in juvenile IIM, especially in juvenile dermatomyositis (JDM), with frequencies ranging approximately 20–40% in JDM, while developing in up to 20% of adult dermatomyositis (DM) [1–5]. Due to the rarity of disease, pathogenesis of JDM-associated calcinosis remains poorly understood, postulated to be an interplay between immune regulation, local vascular ischemia, and intracellular accumulation of calcium due to trauma/inflammation. It has become increasingly important to conduct early screening and routine assessment to avoid complications of calcinosis including pain, skin ulceration, and superimposed infection. Early aggressive treatment has been suggested to prevent calcinosis development [6, 7], however, the true incidence of JDM-associated calcinosis over time has been difficult to elucidate. As new therapies continue to emerge, having standardized clinical response criteria in calcinosis is needed to study the impact of these treatment options. [1, 8].

Calcinosis often emerges in areas of pressure (e.g., hamstrings and gluteus muscles) and repetitive use (e.g., elbows, knees, and wrists), and can be visible to naked eyes when close to the superficial layers of the skin. However, it may also occur in muscles, along fascial planes and around internal organs, which can remain undetected despite a comprehensive physical exam [2, 9]. Understanding the full extent of calcinosis burden and response to therapy remains a challenge as most pediatric rheumatologists reported using only history and/or physical exams to screen for calcinosis in JDM patients [1]. Currently, there are several imaging modalities available to evaluate calcinosis, including X-rays, computed tomography (CT), magnetic resonance imaging (MRI), and ultrasound (US). In a survey of 118 providers in the Childhood Arthritis and Rheumatology Research Alliance (CARRA), X-ray was the initial imaging study obtained in 88% of JDM cases evaluated for possible calcinosis, followed by US in 18%, MRI in 17% and CT in 4% [1]. After calcinosis detection, 95% of those respondents used a physical exam to monitor the response of the calcinosis to therapy, whereas 70% also used additional imaging. Of those using imaging to following-up clinical response, X-rays remained the imaging modality of choice in 72%, followed by MRI in 15%, US in 7%, and CT in 4% [1].

Despite increasing awareness regarding the importance of utilizing these imaging modalities, there is no standardized recommendation on optimal usage in evaluating IIM-associated calcinosis. We provide a narrative review on each imaging modality used to evaluate IIM-associated calcinosis, with a particular focus on evidence in JDM.

## Main text

### Search strategy

A comprehensive literature search was conducted in July 2024 via MEDLINE/PubMed using the following keywords and their Medical Subject Headings (MeSH) terms: “juvenile dermatomyositis,” “dermatomyositis,” “calcinosis,” “radiograph,” “computed tomography,” “magnetic resonance imaging,” and “ultrasound.” Boolean operators (AND, OR) were used appropriately for each imaging modality [10]. The search was performed in July 2024 and covered the period 1967–2024. Clinical trials, observational studies, case series, and case reports reporting use of imaging modality in assessment of DM or JDM-associated calcinosis were included in the review process. There were over 250 articles initially reviewed, of which those that are not in English or not providing description of the imaging modalities were excluded. Ultimately, 56 articles were selected for this narrative review.

### X-ray

**Fig. 1:**
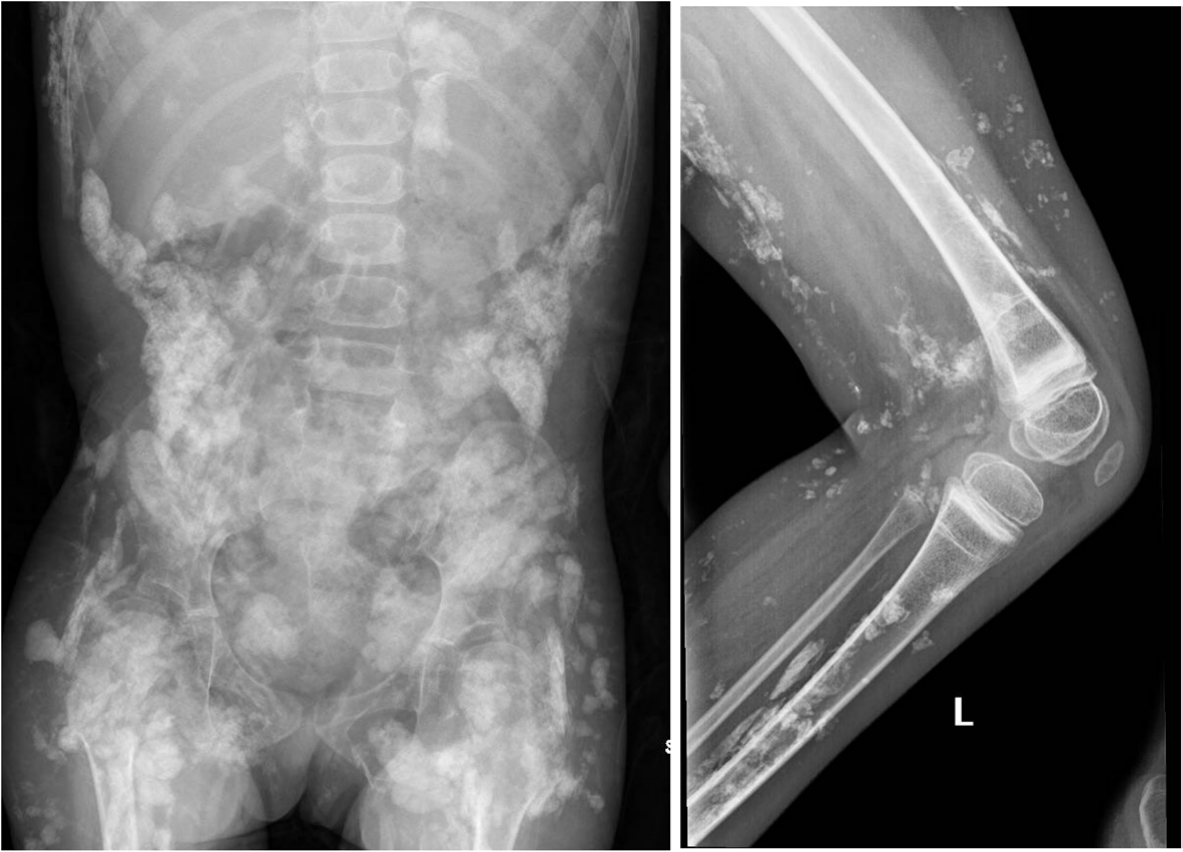
X-ray of abdomen and left knee with extensive amorphous and multilobulated calcinosis in a 7-year-old female with refractory JDM

X-ray is a widely used imaging modality to diagnose calcifications in autoimmune connective tissue diseases such as IIM or scleroderma. X-ray became a modality to diagnose and monitor calcinosis as clinicians recognized the need to detect subcutaneous and intramuscular calcinosis, often missed on physical exam (Fig. 1) [11, 12]. In a retrospective study reviewing 29 JDM related calcinosis cases from 1950–1977, serial radiographs at 1–4-year intervals were able to follow the regression of the subcutaneous calcification in 8 out of 11 children and progression of calcinosis in one child [12]. In another study with 17 patients with dermatomyositis and JDM, radiography was found to be very sensitive and was able to detect calcinosis in all patients [13].

As one of the most common imaging modalities used in evaluating calcinosis, X-ray has been used to describe different patterns of JDM-associated calcinosis. In a case series of 40 patients with JDM, four distinct patterns of calcification were described on X-ray including: deep calcaneal masses, superficial calcaneal masses, deep linear deposits, and lacy, reticular, subcutaneous deposition of calcium encasing the torso [14]. A separate study described the radiological patterns of calcinosis associated with autoimmune connective tissue diseases as nodular (extremity), sheet-like (extremity), reticular (extremity), amorphous (hand), and linear (trunk) [13]. Other studies describe calcinosis found on x-rays as “diffuse palpable subcutaneous nodule,” “periarticular calcinosis with increased opacity,” “calcified nodules in subcutaneous, subfascial, and muscular planes,” “linear and stippled calcification in the subcutaneous tissue,” “generalized superficial calcification with a lacy reticular radiographic pattern,” and “scattered sheets of subcutaneous calcification” to name a few [15–20]. Despite their documented utility in visualizing calcinosis, these studies demonstrate the wide variety of calcinosis distribution and terminology used to describe these lesions.

X-ray remains a useful, accessible, sensitive, relatively low radiation, and widely available modality for diagnosis and monitoring of calcinosis. There is a standardized scoring system to measure calcium deposits in hands for scleroderma patients, but no such scoring system currently exists for IIM patients [21]. Limitations of x-ray include that it is a 2-dimensional exam thus making methods such as US and CT more sensitive for detection of early subtle calcifications as well as localization of the exact anatomical plane and associated complications [22]. In addition, X-rays are typically used in areas where clinical suspicion for calcinosis is noted due to an abnormal physical exam and therefore may miss deeper fascial, non-clinically palpable calcifications unless a whole-body screening skeletal survey is performed. In the Single Hub and Access point for pediatric Rheumatology in Europe (SHARE) guidelines, the expert panel recommended manual palpation and plain radiographs for the detection of calcinosis, with no mention of other modalities due to the lack of scientific evidence [23].

### Computed tomography

**Fig. 2:**
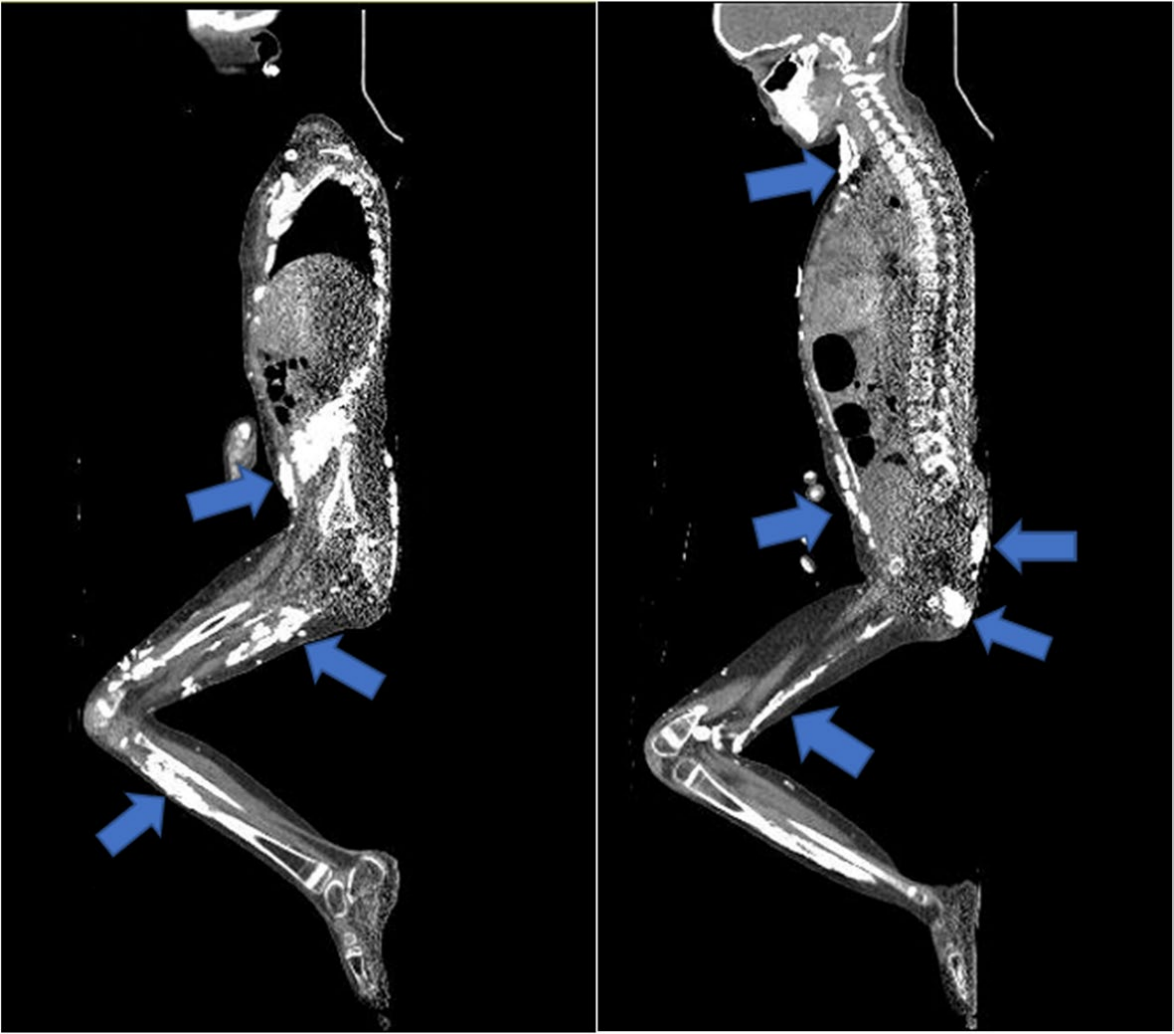
Low-dose whole body CT of a 7-year-old female with refractory JDM and extensive calcinosis universalis. Arrows demonstrate prominent locations of the extensive calcifications in the soft tissues and musculature extending from the neck, along the torso, upper and lower extremities. Also demonstrated are intra-abdominal and pelvic calcifications

CT is used less frequently than X-rays in the assessment of IIM associated calcinosis due to increased risk of radiation, higher cost, and limited access. However, CT provides beneficial spatial information on calcinosis that is difficult to evaluate with one or two-dimensional X-ray images. CT can evaluate lesions that are not readily palpable on the skin, including lesions near internal organs and/or muscles (Fig. 2). CT has been used to image patients since the 1970s [24], with case reports of CT being used to describe calcinosis in dermatomyositis during the 1980s [25, 26]. These early reports highlight cases where CT of the thighs detected prominent calcinosis not previously identified on the skin [25, 26]. Limbs have remained the most common areas assessed with CT, providing vital information on the extent of calcinosis in intramuscular and subcutaneous spaces of extremities [25–31]. In addition, there are several case reports of chest and/or abdominal CT findings of calcinosis in peritoneum, paravertebral muscles, and subcutaneous fat along abdominal wall [32–35]. Most of these cases with internal calcinosis led to escalation of therapy for dermatomyositis or change in management with calcium modifying agents such as bisphosphonates [25, 29, 31–36].

Ibarra et al. reported that using low-dose four-slice CT on affected limbs of JDM patients can provide objective data on the volume of calcinosis and can be a valuable assessment tool without concerns of increased radiation. In this study, the average radiation dose for the CT on upper or lower extremities ranged from 0.007 to 0.01vmSv, compared to a standard chest x-ray amount of 0.02 mSv [28]. More recently, Cervantes et al. utilized low-dose whole-body CT imaging to quantify the amount of calcium in JDM and DM associated calcinosis using the Agatston scoring metric (a numeric score most used to measure calcium deposition in coronary arteries) [37]. The estimated radiation dose was 0.73 ± 0.68 mSv, compared to the standard CT protocol with a radiation dose of 6.32 ± 3.62 mSv. The study showed that patterns of calcinosis can be more accurately subcategorized (clustered, disjoint, interfascial, confluent, and fluid-filled) via CT and that Agatston score may be used as a metric to assess the amount of calcinosis [37].

Another whole-body imaging modality that can be used for screening calcinosis is single-photon emission tomography-CT (SPECT-CT) with planar bone scan, although this method tends to deliver higher radiation doses than the low-dose whole-body CT. SPECT-CT may be useful in patients with multiple areas of calcinosis [36, 38] and still maintains a lower radiation dose than the standard CT protocol, making it a suitable option for pediatric patients. 3D CT reconstruction images are becoming more common and provide an even more accurate assessment of calcinosis [27, 31, 37]. 3D CT visualization uses cinematic rendering, which creates photorealistic images and applies a lighting model that refines surface details and shadowing effects [27]. 3D reconstruction images helped assess the degree of calcinosis in the case of a dermatomyositis patient with dysphagia and orthopnea, where calcinosis was compromising the airway [39].

As technology advances, CT may become a preferential imaging modality for JDM calcinosis with a lower radiation exposure risk. Utilizing 3D reconstruction images will provide even more information on the total burden of calcinosis in patients with JDM.

### Ultrasound

Ultrasonography is a safe, non-ionizing, low-cost and non-invasive radiographic modality that can provide detailed and dynamic information regarding the detection and monitoring of calcinosis in patients with IIM. Literature is sparse regarding the use of US specifically for calcinosis in these patient groups, with justification for usage extrapolated from other autoimmune conditions, such as scleroderma. In one study, sensitivity of US in scleroderma related calcinosis was reported as high as 89% (17 of 19 patients [95% CI: 66%, 97%]) [40], however other studies have demonstrated no significant difference in detection compared to conventional X-ray.

**Fig. 3:**
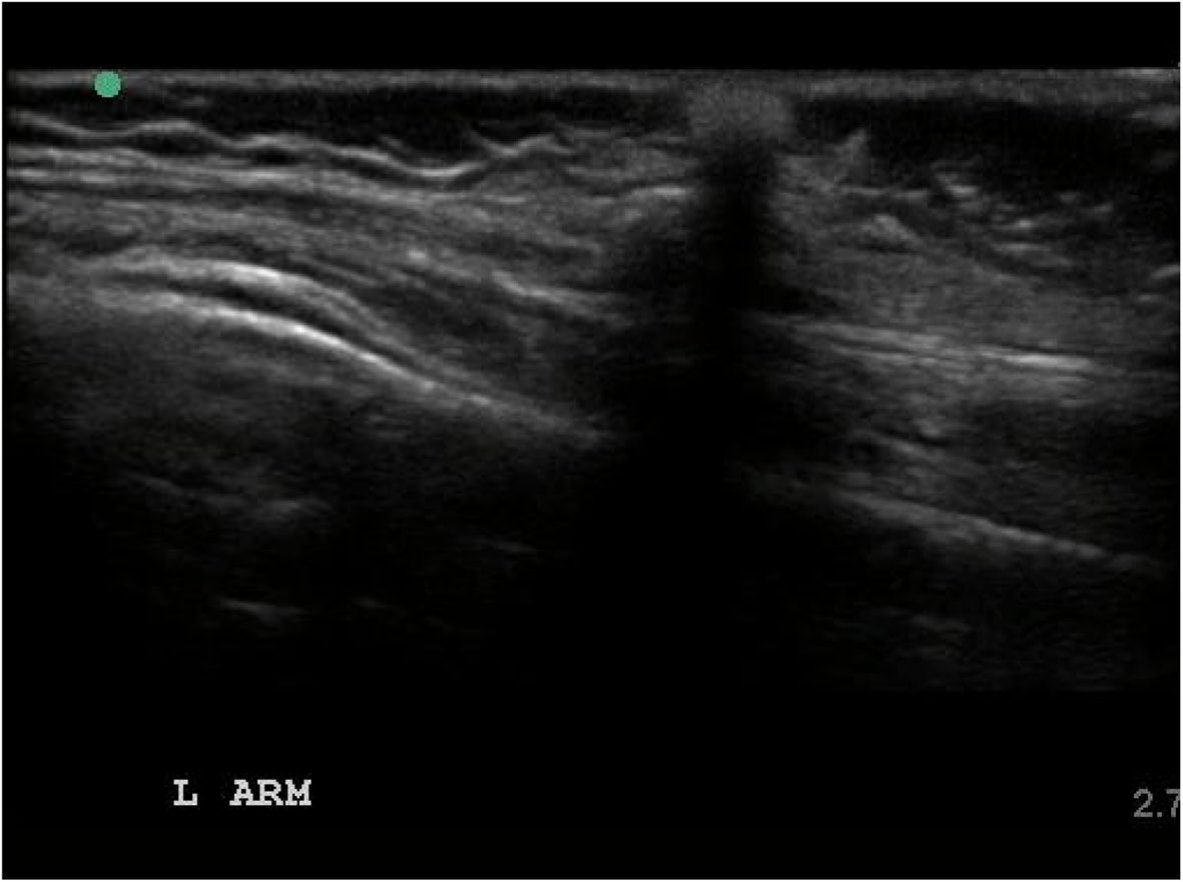
US of left arm with evidence of focal area of hyperechogenicity with posterior acoustic shadowing consistent with subcutaneous calcinosis in a 7-year-old female with refractory JDM

Sonographic evaluation of calcinosis reveals bright, focal areas of hyperechogenicity with posterior acoustic shadowing (Fig. 3), a signal void since the sound waves cannot penetrate the surface of highly dense calcium deposits [41]. In contrast to X-ray, US provides a precise location of the calcinosis within the subcutaneous, intramuscular, and fascial planes, and allows for discrete measuring of calcium deposits for subsequent monitoring. Ultrasonography can also detect various degrees of echogenicity providing a distinct advantage over other modalities to describe not only the location and extent of calcinosis, but also providing information regarding the composition and fluidity. In certain cases of calcinosis, the substance within may reveal a compressible, hyperechoic milky substance that could be misdiagnosed as an abscess [42] and detection of a fluid-calcium layering sign may be useful in the distinction [43]. US additionally allows for doppler imaging to assess vascularity, although caution must be taken not to over interpret a “twinkling artifact” which may occur near the hyperechoic surface and give a false appearance of increased blood flow [44].

Several case reports additionally describe the use of ultrasound to detect calcinosis in atypical locations in patients with JDM and DM including cases of ureteral calcinosis [45], nephrocalcinosis [46], and parotid gland calcifications [47]. In addition to detection and monitoring treatment response, US has also been used to provide visual guidance for focal administration of medications including sodium thiosulfate [48]. Current limitations of US include that it is operator dependent, less reproducible and may be difficult to utilize in detecting an extensive burden of calcinosis.

### Magnetic resonance imaging

High contrast resolution, multiplanar images, and lack of associated radiation make MRI a useful imaging modality for the evaluation of many soft tissue and bony pathologies, including muscle diseases such as IIM [49]. Short Tau Inversion Recovery (STIR) or fat-suppressed, T2-weighted images can be utilized to localize muscle edema/inflammation and can aid in the assessment of muscle disease activity. Additionally, T1-weighted images can demonstrate features of disease damage including fibrosis, atrophy, and fatty infiltration [49].

While not included in the Bohan and Peter or the European League Against Rheumatism (EULAR)/American College of Rheumatology (ACR) diagnostic criteria for inflammatory myopathies, MRI has become an increasingly valuable, noninvasive tool in the diagnosis of JDM [50–52] and has been part of diagnostic guidance per scientific groups such as SHARE and PRAJ (Pediatric Rheumatology Association of Japan (PRAJ)/The Japan College of Rheumatology (JCR) [23, 53].

Despite widespread use in the diagnosis of JDM, MRI has not been reported to be routinely used for calcinosis screening. There are a limited number of case reports that have identified focal and diffuse calcification as well as milk of calcium fluid collections via MRI [46, 54–56]. As MRI is used frequently at the time of IIM or JDM diagnosis, there are studies that have examined whether areas of subcutaneous edema at the time of diagnosis have subsequently led to developing calcinosis with conflicting results [57, 58]. Due to its increasing availability, whole-body MRI is also used to better understand the extent of disease activity at the time of JDM diagnosis [59, 60], although reports using whole-body MRI for calcinosis screening were not found.

**Fig. 4:**
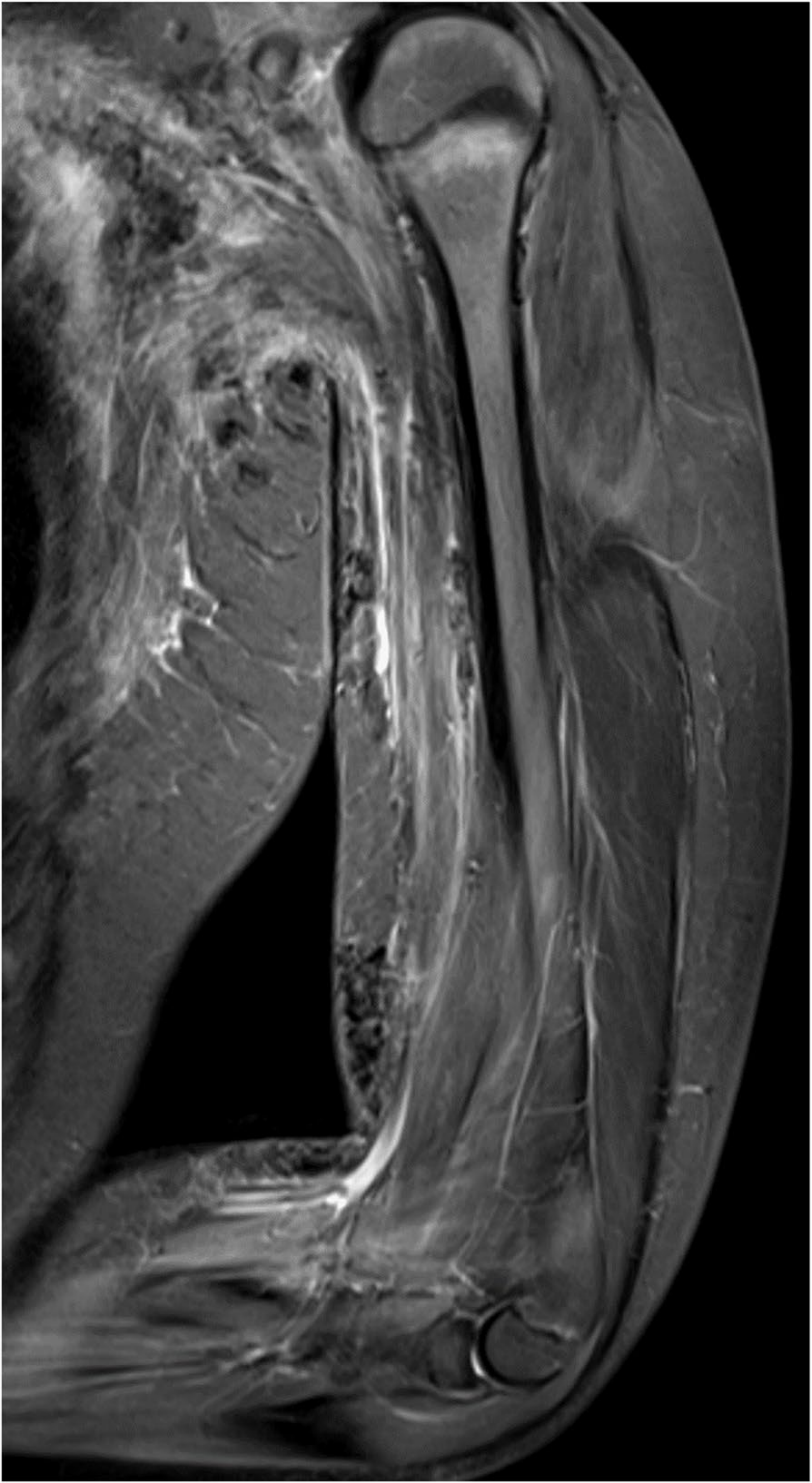
Coronal T2 STIR MRI with multiple areas of hypointensive signal corresponding to extensive lobular subcutaneous calcinosis in a 12-year-old female with juvenile dermatomyositis

In addition to increased cost and inaccessibility, one of the limitations for MRI is that calcification varies in water content and therefore can appear hyperintense, isointense, or hypointense to muscle (Fig. 4) [61]. There are newer echo-gradient techniques such as susceptibility-weighted imaging which can increase the sensitivity and specificity of identifying calcification of soft tissues [62–64]. However, utility of this imaging modality is limited by cost, time, and potential need for sedation in young children. There have been no reports using this technique to screen for JDM associated calcinosis.

As MRI and whole-body MRI are becoming increasingly more important in the diagnosis and monitoring of JDM, additional studies are needed to evaluate their utility in the qualitative and quantitative assessment and longitudinal monitoring of calcinosis over time.

## Conclusions

Calcinosis is a manifestation of IIM, causing pain, disfigurement, infection, and disability. It is more common in the pediatric population, especially in JDM. Unfortunately, there is no standardized treatment for calcinosis, often resulting in long-term complications and increased patient morbidity. Routine screening for calcinosis is important in JDM patients, particularly in those with established calcinosis, to track disease progression. Understanding the true burden of calcinosis in a patient with JDM can be challenging, particularly those with calcinosis located within muscles and near internal organs, thus optimizing the use of various imaging modalities is imperative in the care of these children. However, this remains a challenge as there are no established guidelines on the use of imaging in JDM-associated calcinosis. X-ray remains the most used imaging modality in assessing calcinosis, being low-cost, relatively low radiation, and accessible in most clinical settings. However, its 2-dimensional exam limits complete understanding of the size and volume of calcinosis and involvement of surrounding tissues. CT is available to provide 3-dimensional images, and low-dose whole-body CT and SPECT CT offer detailed imaging with lower radiation risk compared to standard CT protocols, making CT a future option for calcinosis screening. However, the accessibility and cost of the newer technology remains a challenge. US is another low-cost imaging modality that can provide specific location and extent of calcinosis compared to x-rays, but it is operator dependent, and its use is limited in large-sized and internally located calcinosis. Finally, MRI is a valuable tool that can provide high resolution and multiplanar images with lack of radiation. It is not used as frequently as other imaging modalities to assess calcinosis due to its high-cost, time demand, and lower sensitivity for detecting calcinosis. However, new technology using susceptibility-weighted imaging increases MRI’s specificity and sensitivity in detecting calcinotic lesions. Newer technologies are promising, although will need to be accessible in various clinical settings to be used as standardized measures. Further research is needed to better understand different imaging modalities for JDM associated calcinosis.

## Data Availability

No datasets were generated or analysed during the current study.

## Abbreviations

IIM: Idiopathic Inflammatory Myopathies
JDM: Juvenile Dermatomyositis
DM: Dermatomyositis
CT: Computed tomography
MRI: Magnetic resonance imaging
US: Ultrasound
